# Introduction of Induction Heating is too late for older residents with difficulty in handling fire

**DOI:** 10.1590/1980-5764-DN-2021-0074

**Published:** 2022

**Authors:** Yuriko Kato, Jiro Oonuma, Mayumi Suzuki, Kenichi Meguro

**Affiliations:** 1 Tohoku University, New Industry Creation Hatchery Center, Geriatric Behavioral Neurology Project, Sendai, Japan.; 2 Tohoku University, School of Medicine, Sendai, Japan.; 3 Tohoku University, Cyclotron and Radioisotope Center, Sendai, Japan.

**Keywords:** Alzheimer Disease, Cognitive Dysfunction, Induction Heating, Burns, Doença de Alzheimer, Disfunção Cognitiva, Aquecimento por Indução, Queimaduras

## Abstract

Handling errors with household flammables, for example pan burning, may result in serious accidents, which may be caused by decreased attention or executive function.

**Objective:**

The manuals by several cities simply suggest the use of induction heating (IH) cookers. However, it requires complicated operation of buttons. Furthermore, no previous studies have examined the difficulty of IH operation in older adults.

**Methods:**

We examined 166 residents aged 75+ years in Wakuya, consisting 66 Clinical Dementia Rating (CDR) 0 (healthy), 79 CDR 0.5 (very mild dementia), and 21 CDR 1+ (dementia) participants. Based on fire accident, they were classified into “high-risk,” “low-risk,” and “safety” groups. They were asked to actually use an IH as an examination. The participants who passed all procedures were classified as “good users,” and the remaining who failed were classified as “poor users.” Their overall cognitive and executive functions were assessed using the Mini-Mental State Examination (MMSE) and Trail Making Test A and Digit Symbol (DS), respectively.

**Results:**

The proportions of “good users” in the CDR 0, CDR 0.5, and CDR 1+ groups were 7 (10.6%), 6 (7.3%), and 0 (0%), respectively. For the CDR 0 and CDR 0.5 group, the good users had higher scores on the MMSE and DS than do the poor users.

**Conclusions:**

The introduction of IH is too late for “high-risk group.” Since the IH cooker requires complicated operation of buttons, they may be difficult for older residents to handle. Executive function may be examined for early detection of handling errors with household flammables.

## RESUMO.

Erros no manuseio de artigos domésticos muito aquecidos, por exemplo, panelas, podem resultar em acidentes graves, que podem ser causados por diminuição da atenção ou de funções executivas.

**Objetivo:**

Manuais de várias cidades sugerem simplesmente o uso de fogões de aquecimento por indução (FAI), mas esses aparelhos requerem uma operação complexa, com necessidade de uso de diversos botões. No entanto, nenhum estudo anterior examinou a dificuldade de operação de FAI em adultos mais velhos.

**Métodos:**

Foram examinados 166 residentes de Wakuya, Japão, com mais de 75 anos, entre os quais 66 participantes com classificação clínica de demência (CDR) 0 (saudável), 79 CDR 0,5 (demência muito leve) e 21 CDR 1+ (demência). Em função do risco de queimadura, eles foram classificados nos grupos “alto,“ “baixo risco” e “sem risco”. Solicitou-se que realmente utilizassem o FAI como parte do exame. Os participantes que passaram em todos os procedimentos foram classificados como “bons usuários”, enquanto os demais foram classificados como “usuários fracos”. As funções cognitivas e executivas globais foram avaliadas por meio do Miniexame do Estado Mental (MEEM), do Teste de Trilha A e do Teste Dígito-Símbolo (DS), respectivamente.

**Resultados:**

As proporções de “bons usuários” nos grupos CDR 0, CDR 0,5 e CDR 1+ foram 7 (10,6%), 6 (7,3%) e 0 (0%), respectivamente. Para os grupos CDR 0 e CDR 0,5, os “bons usuários” tiveram pontuações mais altas no MEEM e no DS em comparação com os “usuários fracos”.

**Conclusões:**

A introdução de FAI é muito tardia para o grupo de “alto risco”. Uma vez que o FAI requer uma operação complicada com o uso de botões, o seu manuseio pode ser difícil para os residentes mais velhos. A função executiva pode ser examinada para a detecção precoce de erros de manuseio de aparelhos domésticos que podem causar acidentes.

## INTRODUCTION

In Japan, the “New Orange Plan” promotes measures against dementia, suggesting people with dementia to remain in their own home as long as possible. For maintaining their daily life, however, people with dementia or mild cognitive impairment (MCI) face difficulties. They should appropriately manage “fire” and “water,” for ordinary domestic life, because both may likely lead to death when serious accident occurs.

Only humans can use “fire” for living, such as warming and cooking. Fire is thought to be sacred since it is dangerous and could cause a “disaster.” “Small fire accidents” caused by forgetfulness in turning a gas cooking stove off or handling errors may result in pan burning and lead to serious accidents.

According to the report published by the Fire and Disaster Management Agency in 2018, while the number of fire accidents generally tended to decrease annually in Japan, 21,280 building fire accidents still occurred, accounting for 50% or more of all fire accidents. The number one cause of building fire accidents was “stove,” which led to 2,963 (13.9%) accidents. Since failures in handling of fire in households may cause accidents that threaten life, body, and property, appropriate risk management is required.

It was clarified that instrumental activity of daily living (IADL), including cooking, would decrease in persons with MCI or even in healthy elderly persons^1^. We previously reported that accidents caused by the elderly who failed to handle fire, such as pan burning (hereinafter referred to as fire accidents), were related to their executive function, memory, and judgment^2,3^.

Fire accidents may inhibit older residents from continuing to live at home. It is recommended to use electromagnetic cookers (induction heating [IH]) to reduce fire accidents because IH causes less fire accidents compared with direct fire (i.e., the dementia countermeasure manual in Sendai City).

However, we experienced several cases, in which the elderly who were expected to operate IH cookers without fail could not learn the necessary IH operation procedures, as IH cookers require several procedures for use, compared with gas cookers. However, no previous studies have examined IH operation by the elderly.

To investigate the appropriate timing and adaptation of IH introduction, we performed a standard IH operation test, general cognitive function test, and executive function test, to examine their relationships with actual situations in handling of fire in daily life among the elderly.

## METHODS

### Participants

We examined 166 residents aged 75 years or above in Wakuya, northern Japan, which consisted of 66 Clinical Dementia Rating (CDR) 0 (healthy), 79 CDR 0.5 (very mild dementia), and 21 CDR 1+ (dementia) participants (see below). The prevalence rate of dementia was approximately 13%, which was almost equivalent to the national average.

### Clinical Dementia Rating assessment

A clinical team, composed of physicians (neurologists and a psychiatrist) and public health nurses, determined the CDR for each participant and was blinded to the cognitive test results. They used a Japanese version of the CDR scoring questionnaire^4,5^. Before the interviews with the physicians, the public health nurses visited the subjects’ homes to evaluate their daily activities. Observations by family members with respect to the subjects’ lives were described in a semi-structured questionnaire. Subjects who lived alone were visited frequently by public health nurses to evaluate their daily lives. The physicians interviewed the subjects to assess episodic memory, orientation, and judgment. Finally, with reference to the information provided by the family members, the CDR for each of the subjects was determined at a joint meeting of the physicians and public health nurses. One author (KM) was certified as a CDR rater at the Washington University School of Medicine Alzheimer’s Disease Research Center Memory and Aging Project.

### Neuropsychological examinations

For each participant, we performed:

- Evaluation of the ability to operate an actual device (standard IH cooker).
- General cognitive function evaluation (Mini-Mental State Examination [MMSE])^6^.
- Executive function evaluation (Digit Symbol and Trail Making Test A), as well as a questionnaire survey and interview with their families regarding actual fire handling in the households.

### Risk classification (three groups)

The participants were divided into three risk groups for fire accidents based on the incidence of fire accidents during recent 2 years, such as forgetting to put out the fire (e.g., stove, cigarette, or heating device), pan burning, and tatami mat burning, reported by their families, based on the “frequency of small fire accidents at home” and “the presence or absence of pan burning” in the previous 3 years, as follows (Table 1):

- Safety group (n=98): No small fire accident and absence of pan burning.
- Low-risk group (n=39): 0–1 small fire accidents, in the absence of pan burning.
- High-risk group (n=29): one or more small fire accident(s) and the incidence of pan burning, or frequent small fire accidents.

**Table 1:** Risk classification of fire accident.

Pan/tatami mat Burning\Fire accident	Occurred	None
None	5	98
Occurred but the annual incidence is less than 1	8	17
Occurred once to several times per year	24	13
Daily (once or more per month)	4	2

Blue box shows “safety group,” yellow boxes indicate “low-risk group,” and red boxes indicate “high-risk group.”

### Task to operate an actual induction heatingcooker

**Figure 1:**
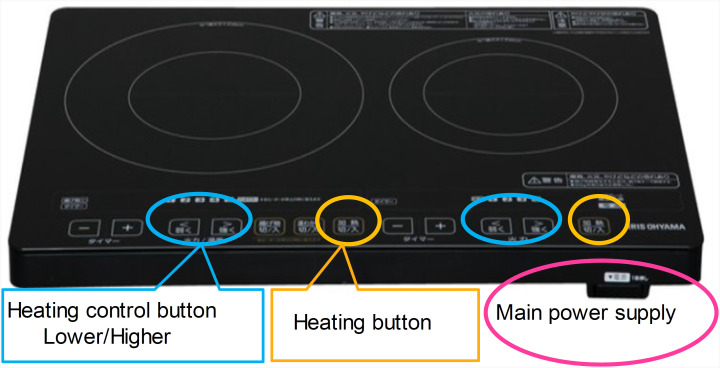
A standard type of induction heating cooker used in the experiment (IRIS OHYAMA induction heating cooking heater, EIH1470/EIH1470V).

Each participant was asked to “boil the water required for cooking pot noodles as soon as possible. When the water is boiled, the kettle will sound, and then turn off the cooker.” The participants were allowed to refer to the IH cooker instruction manual. The participant could repeat the procedure after making a mistake. In addition, when a participant could not operate the cooker by themselves, the participant was allowed to refer to the instruction book prepared by our department (the original manual) and then move on to the next procedure with support from the examiner. In the original manual, a large font (44 pt) was used, as the font was found in our previous report to be clear for the elderly with decreased cognitive function, and the procedures have been described briefly. For the experiment, we selected a standard type of IH cooker (Figure 1).

#### Operation manual

The operation manual includes the following five steps. During each step, we observed a series of actions of the participants to evaluate the situations of their operation.

- Place a kettle on the center of the heating area.
- Turn on the main power supply.
- Start heating by pressing the heating button.
- Increase heat to maximum using the heat control button.
- When the water in the kettle has boiled, stop heating by pressing the heating button again.

### Classification by capability to operate (two groups)

Based on the execution of a task to operate the IH cooker, the participants were divided into two groups as follows:

- Good user group (those who could complete all operation steps from 1 to 5).
- Poor user group (those who failed to operate a step).

### Ethics

An explanation related to the study was held in public halls. After obtaining written consent from each participant, public health nurses visited the participant’s home on another day to obtain consent from family members. This study was approved by the Institutional Review Board of our university (2014-1-565).

## RESULTS

### Capability to carry out the induction heating cooker for the risk groups and IH with the Clinical Dementia Rating levels

**Figure 2:**
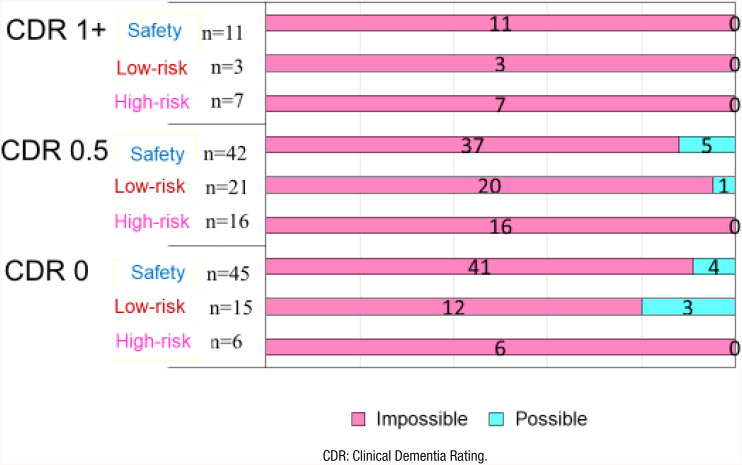
Relationship beteween the risks of fire accidents and Induction Heating operation tasks with the Clinical Dementia Rating levels.

In the healthy group, 15% of the participants could operate an HI cooker, and 85% could not complete the operation by themselves (Figure 2). In the MCI group, approximately 8% could operate an IH cooker, and 92% could not. In the dementia group, no one was able to operate the IH cooker.

Although none of the group differences were significant, it was noteworthy that even in the healthy group, only 15% were able to operate the IH.

### Operation of the induction heating cooker in individual steps

Table 2 shows the details of major errors observed in each of the individual operation steps.

**Table 2:** Details of operation errors.

Operation steps	Details
1) Place a kettle on the center of the heating area of the IH cooker.	– Placed the kettle on an area far from the center circle of the heating area.
2) Turn on the main power supply.	– Could not find the location of the main power supply (e.g. it was difficult to distinguish the location because all parts are black). – Could not press the main power supply, but the part of white characters which suggested the power supply. – Could not press and hold (for 1 second).
3) Start heating by pressing the heating button.	– Pressed the heat control button located on the other side (right/left) by mistake. – Pressed the button for cooking of fried foods.
4) Increase heat level to the maximum using the heat control button.	– Could not set at high power (maximum heat level) (did not understand the meaning of high power). – Pressed the timer button.
5) When the water in the kettle has boiled, stop heating by pressing the heating button	– Pressed the button to “turn off,” but it did not work. – Turned off using the main power supply, not by using the heating button.
*General/other errors	– Could not read the characters. – Pressed the part of the white characters instead of the button. – Could not see the red light. – It was difficult to press the flat button on the flat surface (e.g. because of pain in the hand). – Even when pressing the button, the action could not be sensed by the cooker. – Continued operation even if the cooker did not work. – Could not understand the procedures even after reading the manual.

* It was difficult to press the flat button on the flat surface (e.g. because of pain in the hand). Even when pressing the button, the action could not be sensed by the cooker.

The details of the impressions and ideas for improvement were obtained from 100 participants after they finished the tasks. In spite of their performance in their operation, approximately 40% of the participants answered that the cooker was easy to use, or very easy to use. From difficulty to easy, the ratios of participants selected “very difficult to use,” “difficult to use,” “easy to use,” and “very easy to use” are 5, 22, 37, and 5%, respectively. Also, 25% selected “cannot say,” and no data for 6%.

Free descriptions by those selected “very difficult to use” and “difficult to use” are:

- It is difficult to use the cooker because there are several buttons. Single action is better.
- A flick of a switch would be better.
- A larger display would be better.
- All displays appear to be similar.
- The power supply button should be located close, next to (or just above) its display, as it is difficult to find it.
- It is difficult to recognize the display for turning on/off, and thus large characters indicating ON and OFF should be provided.
- The buttons should be located at an area other than that on the superior surface.
- Flat buttons are difficult to use, and conventional in-and-out buttons are better.
- It is better for a user to clearly feel the pushing action.
- There are no buttons to stop or suspend heating.
- The elderly cannot understand “high power.”
- It is difficult to find the light (red), and thus a larger light should be used.
- It is better that the cooker has a speaking function.
- I feel safe to visually confirm the heat/fire, as it is difficult to confirm whether or not the IH cooker is working.

Free descriptions by those selected “easy to use” and “very easy to use” are:

- IH will be easy to use when I get used to it.
- IH seems like it will be easy to use after I learn how to use it.
- IH may be better to ensure safety.
- Since the cooker is flat, it appears to be stable.
- IH is good as it is easy to clean.
- IH is sophisticated.

### Relationship between the induction heating operation and neuropsychological test results

Table 3 shows the mean scores of MMSE, DS, and TMT-A between the possible/impossible IH operation healthy and MCI groups. The MMSE and executive function (DS) were significantly higher in the group with possible IH operation, compared with the group that could not operate the IH cooker. No significant difference was observed between IH cooker capabilities regarding executive function (TMT-A).

**Table 3:** Relationship between the induction heating operation and neuropsychological test.

CDR 0+0.5 n=154	IH operation task			
	Possible n=18		Impossible n=136	
	mean	SD	mean	SD
MMSE	26.1	2.9	24.5*	3.1
DS	35.1	11.4	29.7*	8.8
TMT-A	54.0	25.5	62.0	21.9

CDR: Clinical Dementia Rating;

* p<0.05;

degree of freedom=95; statistical analysis: Student’s t-test; Possible vs. Impossible; MMSE: Mini-Mental State Examination; DS: digit symbol; TMT-A: Trail Making Test A.

## DISCUSSION

Our investigations showed that even in the healthy group, there were “high-risk” people. In all of the CDR groups, the high-risk group was not able to manipulate the IH cooker. In the dementia group, even the “low-risk” people were not able to manipulate the IH cooker. Furthermore, 17% of the participants in the MCI/low-risk group were able manipulate the IH cooker, compared with 40% in the healthy group. These individuals also showed higher scores on the MMSE and the executive function tests.

Executive function is the overall ability to control, including attention, and may be specific to risk control. As mentioned in our previous study^3^, the descriptions of fire accidents showed that the accidents were caused by a decrease in attention/executive function and the ability to predict risks in all CDR groups. If the subjects recall that they are cooking without burning a pan, even if they are distracted by something, the behavior could be based on their past memory. Also, controlling manipulation is one of the executive functions. Since the IH cooker requires complicated manipulation of the button, they may be difficult for older residents to handle. The error analysis findings suggest that IH cookers could be improved in order to be successfully manipulated by older adults.

Household electric appliances increase the convenience and quality of life (QoL). With further increases in aging estimated in the future, it is expected that further technical developments will improve household electrical appliances so that the elderly who are not good at handling such appliances due to lowered cognitive and physical functions can receive the benefits.

It was considered that the reason for their preference of the use of a cooking stove was that the switch system was easy for them to handle. While IH appliances are recommended for people who find it difficult to handle flammable items, the operation is complicated and not necessarily suitable for elderly. The elderly may have increased burden in learning how to use a new cooking apparatus (tool) with no direct view of the fire and no sensation of heat, because their semantic memory is different from that for conventional tools. The flat work surface, holding down of a button, and other new operational procedures may be inappropriate for the elderly. It may be desirable to construct a cooking cabinet and work surface that would enable easy understanding of ON and OFF using visual, auditory, and tactile feedback. It should be investigated whether the safety, functionality, and ease of use of such a new apparatus would be appropriate for the elderly from the viewpoint of brain function.

It was suggested that the recommendation for introduction to IH cooking would be too late for the “high-risk group,” in which forgetting to put out the fire and pan burning increased. IH should be introduced as early as possible, but even in the healthy group, 85% were not able to operate an IH cooker in this study. However, one limitation of this study is that we did not evaluate the learning effect of the participants. In addition, if a user could not use the IH cooker immediately after purchase of the cooker, the user could not actually cook. In such cases, unexpected accidents could occur, as seen in past reports on fire accidents, that were caused because an elderly person had no other choice but to use an emergency portable gas stove, as the person could not use an IH cooker. To use an IH cooker, general cognitive function and executive function should be maintained. It was suggested that the operating procedures for IH would be difficult for the elderly, although such procedures appeared to be easy.
